# Biological Reactions to Metal Particles and Ions in the Synovial Layer of Mice

**DOI:** 10.3390/ma13051044

**Published:** 2020-02-26

**Authors:** Xiangyun Cheng, Sabine C. Dirmeier, Sandra Haßelt, Andrea Baur-Melnyk, Jan Philippe Kretzer, Rainer Bader, Sandra Utzschneider, Alexander C. Paulus

**Affiliations:** 1 Department of Orthopedic Surgery, Physical Medicine and Rehabilitation, University Hospital of Munich, Ludwig-Maximilians-University, Campus Großhadern, Marchioninistraße 15, 81377 Munich, Germany; xiangyun-cheng@outlook.com (X.C.); Sabine.Dirmeier@gmx.de (S.C.D.);; 2 Department of Radiology, University Hospital of Munich, Ludwig-Maximilians-University, Campus Großhadern, Marchioninistraße 15, 81377 Munich, Germany; 3 Laboratory of Biomechanics and Implant Research, Clinic for Orthopedics and Trauma Surgery, Heidelberg University Hospital, Schlierbacher Landstrasse 200a, 69118 Heidelberg, Germany; 4 Biomechanics and Implant Technology Research Laboratory (FORBIOMIT), Department of Orthopaedics, Rostock University Medical Center, Doberaner Straße 142, 18057 Rostock, Germany

**Keywords:** inflammation, cytokines, metal particles, metal ions, synovium

## Abstract

Metal particles and ions released from implants not only have a fundamental effect on the longevity of total joint replacements, but can also be disseminated to remote organs. Periprosthetic tissues harvested during revision surgeries mainly reflect end-stage failure but may not adequately reveal initial biological reactions and systemic side effects. Therefore, primary reactions caused by metal particles and ions were investigated in an established murine model. Left knee joints in three groups, each consisting of ten female BALB/c mice, received injections of metal ions (MI), metal particles (MP) and phosphate-buffered saline (PBS) (control). Seven days after the injection, immunohistochemical analyses of the synovial layer were performed with respect to some biological markers including Tumor necrosis factor -α (TNF-α), Interleukin-6 (IL-6), Interleukin-1β (IL-1β), Cluster of Differentiation 45 (CD45), Cluster of Differentiation 68 (CD68) and Cluster of Differentiation 3(CD3). The MP group showed significantly enhanced proinflammatory cytokine expression (TNF-α, IL-6 and IL-1β) compared with the other groups (*p* < 0.05). Interestingly, CD3, as a marker for T lymphocytes, did not increase in any of the groups. The MI group showed a significantly increased expression of CD45 compared with the control group (*p* < 0.05). Therefore, during the primary process, metal particles have stronger pro-inflammatory potential than metal ions, and T lymphocytes did not seem to be activated in our murine model. Systemic reactions caused by metal particles and ions were found by observing the untreated right knees.

## 1. Introduction

Medical cobalt-chromium-molybdenum (CoCrMo) alloys are mainly classified by the International Organization for Standardization (ISO) or American Society for Testing and Materials (ASTM) [1] and can be of a cast (ASTM F75, ISO 5832-4) or wrought content (ASTM F1537, ISO 5832-12) [2,3]. Due to their excellent biocompatibility and mechanical properties, these alloys have been widely employed in implant devices that replace hard tissue in the human body [4]. Since the mid-1980s, over one million metal-on-metal (MoM) hip endoprostheses made from a CoCrMo alloy have been implanted worldwide [5]. However, during the 2000s, issues of aseptic loosening due to the release of metal particles and ions were found, and the use of MoM replacements was almost completely stopped [6]. Subsequently, in joint arthroplasties, hybrid combinations were mainly recommended, such as a polyethylene inserts and CoCrMo heads (MoP). Unfortunately, metal wear particles and released metal ions have also been found in patients with MoP prostheses due to the mechanically assisted crevice corrosion of modular taper junctions, including the head–neck and neck–stem taper interfaces [7,8]. Some related studies have been conducted, and researchers have found that CoCrMo particles in revision patients are quite small (< 60 nm) and numerous. In general, high levels of metal ions are also generated from MoM implants [9]. Undoubtedly, the generation of degradation products and the subsequent biological reactions to the metal particles and ions have a fundamental effect on the longevity of total joint replacement [10]. However, to date, the effects of small wear particles and metal ions on local or systemic biological reactions remain complex and are still not fully understood in detail. 

Commonly, metal debris, numerous inflammatory cells and increased proinflammatory mediators, such as IL-1β, TNF-α and IL-6, are found in periprosthetic tissues from patients with aseptic loosening [11]. Additionally, a synovial-like membrane with aggressive granulomatous lesions, in which many macrophages are infiltrated, is usually observed around aseptically loosened MoM implants [12]. Therefore, many researchers believe that small metal particles and high levels of metal ions cause an aseptic inflammatory response, which probably leads to periprosthetic osteolysis and, eventually, results in aseptic implant loosening [13]. However, most of the histological evidence for aseptic inflammatory reactions in periprosthetic tissues is from tissues of revision surgeries [14,15], which might not reflect the important initial reactions caused by metal particles and ions. Meanwhile, in terms of metal particles and metal ions, it is still unclear which factor plays a more critical role in aseptic inflammatory reactions.

In addition to aseptic inflammatory reactions, it has been speculated for a long time that type IV hypersensitivity reactions via T lymphocyte activation could play a role in aseptic loosening [13,16]. The assumption is mainly based on the presence of T lymphocytes in periprosthetic tissues from some studies and on the ability of metal ions to activate type-IV hypersensitivity by acting as haptens [17]. However, reactions against metal haptens are mainly determined by individual hereditary factors, with only some patients being genetically susceptible to developing type IV hypersensitivity against metals. Additionally, in some studies, patients with metal-on-metal implants displayed a significant decrease in the number of T lymphocytes and a significant increase in the level of metal ions after the hip replacement [18]. Therefore, it is still somewhat controversial whether type IV hypersensitivity reactions contribute to the aseptic loosening induced by metal particles and metal ions, especially in the short term after joint arthroplasties.

Primary biological reactions around periprosthetic tissues need to be precisely elucidated, not only to improve diagnoses but also to provide valid evidence for subsequent treatment [19,20,21]. Therefore, the objective of the present study was to evaluate and compare the primary aseptic inflammatory reactions to metal ions and wear particles in an established murine model. In addition, to better understand the role of type IV hypersensitivity reactions caused by degeneration products, T lymphocytes were also evaluated in the established model. The first hypothesis is that the metal particle (MP) group and metal ion (MI) group can both induce enhanced proinflammatory cytokine expression (TNF-α, IL-6 and IL-1β), and that T lymphocytes would be recruited when particles and ions were released in vivo. The second hypothesis was that the MI group have a stronger pro-inflammatory potential and recruit more T lymphocytes than the MP group.

## 2. Materials and Methods

### 2.1. Metal Particle/Ion Generation 

Standard wrought CoCrMo alloys (ISO 5832 -12, ASTM F1537) [20] for hip implants were used to generate metal ions and wear particles in this study. 

To obtain metal particles that were similar to clinically produced particles, a newly developed pin-on-disc simulator was used to perform wear tests at a frequency of 1 Hz. The wear volume was determined in the test medium by a high resolution inductively coupled plasma mass spectrometry (HR-ICP-MS) instrument (Thermo Scientific, Bremen, Germany). After performing an analysis via scanning electron microscopy (SEM; Zeiss EVO 50, Carl Zeiss NTS GmbH, Oberkochen, Germany), the following parameters were recorded: the equivalent circular diameter (ECD), the aspect ratio (AR) and the roundness (R) of wear particles.

To induce the release of metal ions, the CoCrMo alloys were anodized in a corrosion chamber and phosphate-buffered saline (PBS) was used as the surrounding medium. Using an HR-ICP-MS instrument (Thermo Scientific, Bremen, Germany), a total metal ion content (cobalt, chromium, molybdenum and nickel) of 20.5 mg/L was determined, which was adjusted to the desired target concentration of 200 μg/L using PBS. The target concentration of 200 μg/L was based on a study in which the metal ion level of a joint puncture of patients was analyzed prior to revision surgery, which showed average concentrations in the range of 200–250 μg/L in the joint fluid [22]. 

### 2.2. Elimination of Endotoxin

To avoid the influence of adherent endotoxins on the results, the generated particles and ions had to be free of endotoxins. To remove endotoxins, metal particles were cleaned by an ethanol washing process, and the metal ion solution was heat shocked. The elimination of endotoxins was proven by the Limulus Amebocyte Lysate (LAL) test (Lonza, Cologne, Germany).

### 2.3. Animals and Intraarticular Injection

Forty female BALB/c mice (Charles River Wiga Company, Sulzbach, Germany), weighing 23.5 ± 1.9 g, were used to establish an animal model in which biological reactions could be evaluated. These mice were randomly assigned to three groups: the PBS control group (*n* = 10), MP group (*n* = 10) and MI group (*n* = 10). To exclude the possibility that the process of the intra-articular injection itself produces an inflammation reaction in subsequent experiments, the remaining untreated mice (*n* = 10) were regarded as the negative control (NC) group. All experimental steps involving animals were performed according to the rules and regulations of the Animal Protection Laboratory Animal Regulations (2013) and European Directive 2010/63/EU Act, which is in accordance with the National Animal Protection Law (protocol number 55.2-1-54-2532-82.12, Government of Bavaria, Germany).

Prior to the intra-articular injection, all solutions were sonicated for at least 60 min to prevent possible agglomeration and precipitation. Under sterile conditions, 50 μL PBS-suspension, 50 μL of a 0.1 vol% CoCrMo particle suspension and 50 μL of 200 μg/L CoCrMo ions were injected into the left knees of the mice. After a 7-day incubation period, animals were euthanized by an intracardial pentobarbital injection (Merial GmbH, Hallbergmoos, Germany). 

### 2.4. Immunohistochemistry

Both knees (left treated and right untreated) of all groups were removed and fixed in 4% paraformaldehyde for 24 h. All knee joints were decalcified using Osteosoft solution (Merck KGaA, Darmstadt, Germany) for four days at room temperature. Decalcification of the knees was followed by dehydration in a Spin Tissue Processor-120 (Especialidades Médicas Myr, S.L., Tarragona, Spain) in an ascending alcohol series (7%, 96%, 100%, and xylene) and, finally, a transfer to two successive paraffin baths at 60 °C. After being embedded in paraffin, solidified blocks were cut into 2 μm thick sections. Subsequently, the prepared tissue sections were immunochemically analyzed using six different monoclonal mouse antibodies: TNF-a (1: 200 dilution), IL -1b (1: 150 dilution), IL -6 (1: 200 dilution), CD68 (1: 100 dilution), CD45 (1: 400 dilution) and CD3 (1: 100 dilution) (Biorbyt Ltd., Cambridge, United Kingdom). Factors from the spleen (TNF-a), lung (IL -1β and IL-6) and tonsil (CD68, CD45 and CD3), which always react positively with the corresponding antibodies, served as positive controls in our study (data not shown). 

As shown in many studies, the cytokines TNF-α, IL-1β and IL-6 are important inflammatory triggers in peri-implant tissues and are even involved in subsequent prosthetic loosening [23,24]. Therefore, in this study, TNF-α, IL-1β and IL-6 were used as inflammatory markers. According to the pro-inflammatory cytokine expression (TNF-α, IL-6 and IL-1β) in immunohistochemistry, we assessed the degree of inflammatory response in each group.

CD45 can be expressed in various immunocompetent cells, including dendritic cells, T lymphocytes, B lymphocytes, macrophages, etc. [25,26]. CD45 was used as a general marker of immunocompetent cells in the synovial layer in this study. The CD3 antigen binds to the membranes of all T lymphocytes and virtually no other cell types, which makes it a useful immunohistochemical marker of T lymphocytes in tissue sections [27]. The CD68 antigen is mainly expressed by monocytes and macrophages. When monocytes migrate into local tissues, they differentiate into macrophages [28,29]. Therefore, we used CD68 as a marker of macrophages in this study. To analyze the systemic biological reactions caused by metal particles and metal ions in our murine model, the immunohistochemical markers mentioned above were analyzed in the right knees of all groups.

Images of the stained samples were collected under a light microscope at 200× magnification (Carl Zeiss Micro Imaging GmbH, Oberkochen, Germany). Every image included the most synovial tissue (region of interest) of each sample. If cells or tissues were stained from light yellow to brown, positive immunostaining was recorded. Two observers independently performed manual counts of the obtained images with the assistance of Image J software (National Institutes of Health, Bethesda, MD, USA) (Ver.1.43, available at rsbweb.nih.gov/ij). Preceding manual counting, images were cropped, scaled to μm and separated by a color channel, and artifacts were removed. An area tool was used to select the synovial region and calculate the area. The Image J cell counter tool recorded mouse clicks on cells that were labeled with colored dots. The results were saved to a spreadsheet and screen shots were used to record the session. Positive cells from the synovial membrane were counted by each observer. If the results were inconsistent, specific samples were collected and counted by both observers for a third time.

### 2.5. Statistics

The data obtained from the immunohistochemical evaluation were evaluated using IBM SPSS® Statistics 22 (IBM Deutschland GmbH, Ehningen, Germany). Statistical analyses were carried out with the non-parametric Kruskal–Wallis test for independent samples. *P*-values below 0.05 were considered statistically significant and were adjusted by Bonferroni correction. A graphical presentation of the results was produced using box plots.

## 3. Results

### 3.1. Characterization of Metal Particles and Ions

For the generated metal particles, the mean size was in the nanometer range (ECD: 61.25 ± 18.47 nm). Meanwhile, their aspect ratio was 1.69 ± 0.66 and roundness was 0.64 ± 0.16 (Table 1). The particle shape was predominantly round and oval; in addition, a small proportion of acicular particles were formed (shape: round, 44%; oval, 49%; needle, 7%). This size and morphology of the particles were consistent with clinically found metal particles after a MoM total hip replacement [30,31].

Using HR-ICP-MS, a total metal ion content (cobalt, chromium, nickel and molybdenum) of 20.5 mg/L was determined, which was adjusted to the desired target concentration of 200 μg/L using PBS (Table 2) [32]. The target concentration of ions was based on the concentration measured in the synovial fluid of patients with endoprosthesis during revision surgery [22].

### 3.2. Results of the Left Knee Joints

#### 3.2.1. Expression of TNF-α, IL-1β and IL-6 

**Figure 1:**
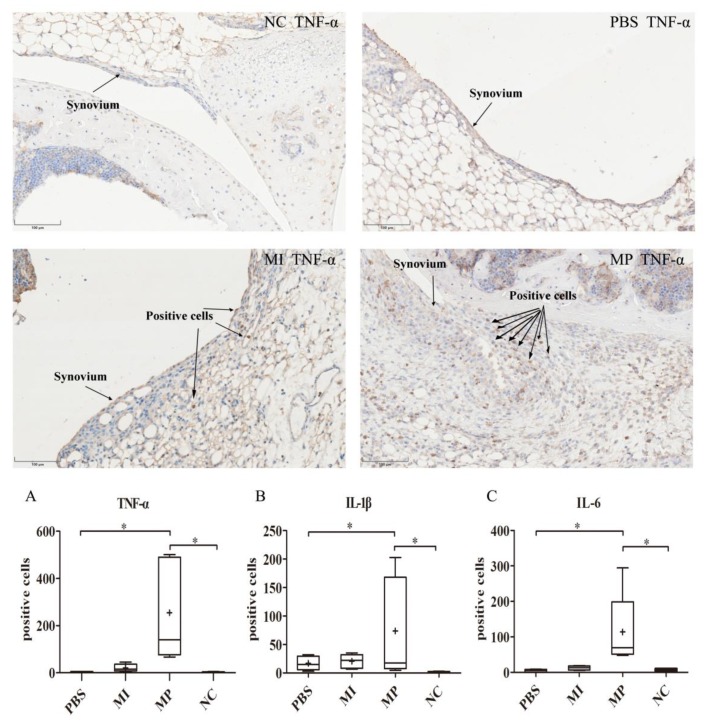
Expression of inflammatory markers (IL-1β, IL-6 and TNF-α) in the synovial layer of left murine knees. The staining results of TNF-α in the negative control (NC), phosphate-buffered saline (PBS), metal ion (MI) and metal particles (MP) group. Both the strongest expression and a thickened synovial layer are found in the MP group. (**A**) The expression of TNF-α in the synovial layer of left murine knees. (**B**) The expression of IL-1β in the synovial layer of left murine knees. (**C**) The expression of IL-6 in the synovial layer of left murine knees. The staining results of IL-1β, TNF-α and IL-6 are consistent. The MP group had the strongest inflammatory reactions. (Magnification: 200x; * *p* < 0.05).

With regard to the staining results of TNF-α, IL-1β and IL-6, the MP group showed a significantly increased number of positive cells compared with the PBS group (*p* < 0.05). However, the MI group did not express significantly more TNF-α (*p* = 0.056), IL-1β (*p* = 0.420) and IL-6 (*p* = 0.124) than the PBS group. Therefore, according to these results, the MP group had stronger inflammatory reactions in the synovial layers of the left knees (Figure 1).

#### 3.2.2. Expression of CD68, CD3 and CD45

**Figure 2:**
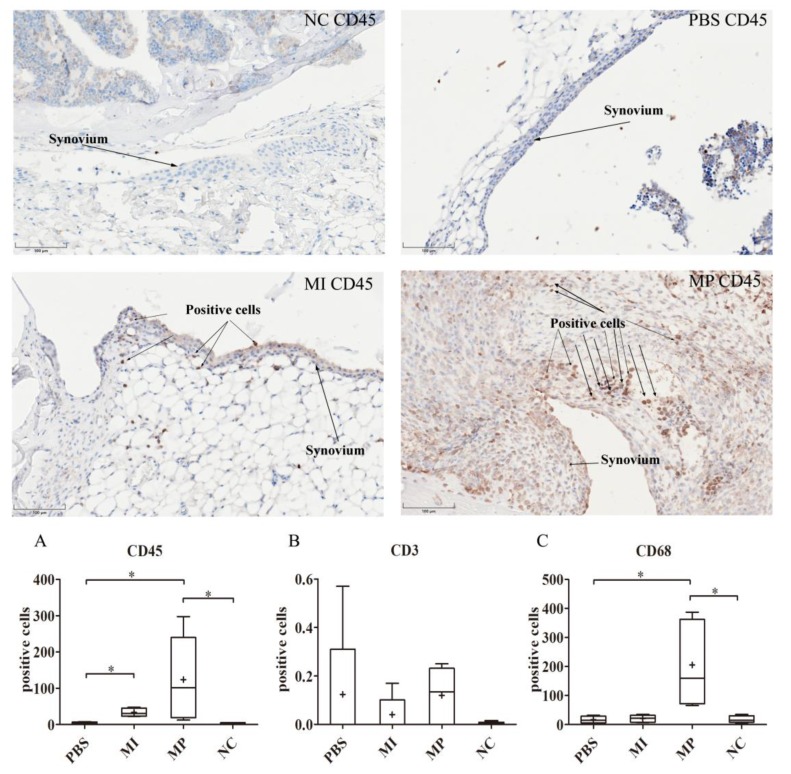
Expression of CD45, CD3 and CD68 in the synovial layer of left murine knees. The staining results of CD45 in the NC, PBS, MI and MP group. Numerous CD45 positive cells were found in the MP and MI group. MP and MI groups had significantly increased positive cells compared with the PBS group (*p* < 0.05). (**A**) The expression of CD45 in the synovial layer of left murine knees. (**B**) The expression of CD3 in the synovial layer of left murine knees. No significant difference was found in all groups. (**C**) The expression of CD68 in the synovial layer of left murine knees. (Magnification: 200x; * *p* < 0.05).

The MP group showed a significantly higher number of CD45-positive cells compared with the PBS group (*p* < 0.05). Likewise, the MI group had a significantly different number of CD45-positive cells compared with the PBS group (*p* < 0.05). Interestingly, in terms of CD45, there was no significant difference between the MP group and the MI group (Figure 2A).

With respect to the expression of CD3 positive cells in our study, there was no statistically significant difference among the three groups (*p* = 0.45) (Figure 2B).

The MP group showed a significantly increased number of CD68 positive cells compared with the PBS group (*p* < 0.05), and the MI group (*p* < 0.05) in the synovial layer of the left knees. There was no significant difference between the MI group and the PBS group (*p* = 1.0). Considering that macrophages play an important role in inflammatory reactions, these results indicate that the MP group had the strongest inflammatory reactions, which is consistent with the TNF-α, IL-1β and IL-6 results (Figure 2C).

### 3.3. Results of the Right Knee Joints

#### 3.3.1. Expression of TNF-α, IL-1β and IL-6 

**Figure 3:**
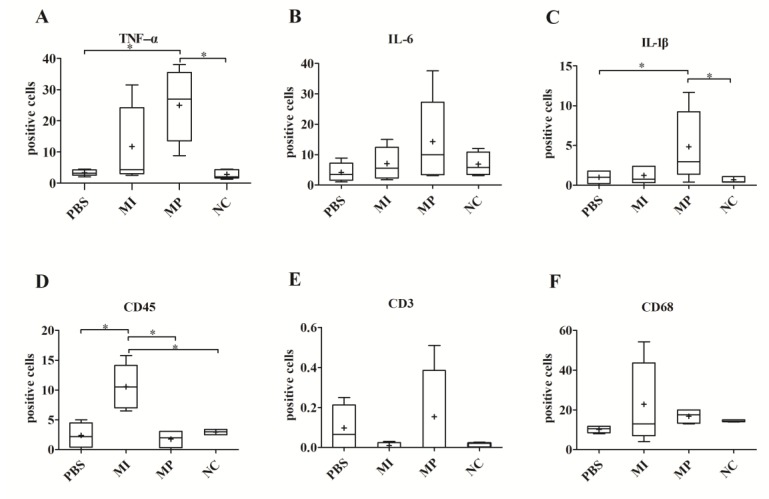
Expression of all biological markers in the synovial layer of right murine knees. The aim of examining the right knees was to evaluate systemic reactions caused by the dissemination of metal particles and metal ions. (**A**) The expression of TNF-α in the synovial layer of right murine knees. (**B**) The expression of IL-6 in the synovial layer of right murine knees. (**C**) The expression of IL-1β in the synovial layer of right murine knees. (**D**) The expression of CD45 in the synovial layer of right murine knees. The MI group exhibited a significant difference compared with the PBS groups (*p* < 0.05). (**E**) The expression of CD3 in the synovial layer of right murine knees. (**F**) The expression of CD68 in the synovial layer of right murine knees. (* *p* < 0.05).

For TNF-α and IL-1β, the number of positive cells in the MP group was only significantly increased compared with the PBS group (*p* < 0.05). There was no significant difference among all groups in the expression of IL-6 in the synovial layer of the right murine knee joints, (*p* = 0.13) (Figure 3A–C).

#### 3.3.2. Expression of CD68, CD3 and CD45

The expression of CD45-positive cells in the MI group was significantly increased compared with all other groups (PBS: *p* < 0.05, MP: *p* < 0.05). No statistically significant difference was found in the direct comparison of the PBS and MP groups (*p* = 1.0). Meanwhile, there was no significant difference among all groups in the expression of CD68 and CD3 in the synovial layer of the right knees (CD68: *p* = 0.55, CD3: *p* = 0.91) (Figure 3E–G).

## 4. Discussion

Our initial hypothesis for this study could not be proved. No increased T-lymphocyte markers were found in either the MP or MI group. However, in terms of proinflammatory reactions (IL-1β, IL-6 and TNF-α), the MP group had stronger reactions than the MI group. In addition, in terms of systemic reactions, the MP group and the MI group both had significantly increased biological reactions in the synovial membrane of right-sided knee joints compared to the PBS (control) group.

Histopathological studies can decisively contribute to the determination of the main cell populations underlying the biological mechanisms of aseptic inflammation and osteolysis [33]. In addition, the key protein molecules (such as IL-1β, IL-6 and TNF-α) involved in aseptic loosening can also be precisely detected by immunohistochemistry [34,35]. Therefore, periprosthetic tissues harvested during revision surgery, especially the bone–implant interface membrane and pseudo-synovial tissues, are frequently examined via histological techniques in clinical work [14,36]. However, periprosthetic tissues from revision surgeries mainly reflect end-stage failure and may not adequately reveal the primary biological reactions caused by metal particles and ions released immediately after the initial surgery. Nevertheless, the primary biological reactions caused by metal products in vivo may be crucial to elucidate the evolution of the pathophysiological events that lead to prosthetic osteolysis. In light of the limitations mentioned above, an established murine model was used in this study, which mimics the initial biological reactions in the synovial-like tissues around prostheses caused by metal particles and ions [35,37,38,39]. In contrast to various in vitro cell culture studies that focus on one cell type [32,40], the murine model can not only reflect complex cellular and tissue interactions, but can also mimic the dynamic process of joints. Additionally, compared with other models, such as hamsters’ skinfold-chamber models [41] and "air pouch" models [42], results from this model, as the suspensions were injected intraarticularly, are easier to translate into a clinical scenario because the generated wear debris primarily accumulates in the joint that has been replaced. In terms of the characteristics of metal materials, the CoCrMo particles and ions used in our study were consistent with those found in some clinical studies. 

CD45 can be expressed on many types of cells in the immune system, such as lymphocytes, natural killer cells, granulocytes, dendritic cells and monocytes/macrophages [26,43]. Therefore, CD45 was chosen as a general marker of immunocompetent cells in the synovial layer. For the left (treated) mice knees, the MP group and MI group both showed significantly increased CD45-positive cells compared with the PBS group, assuming that some immunocompetent cells were recruited to the synovial layer as a result of the metal particles and metal ions. However, it is difficult to distinguish specific immunocompetent cells and biological reactions solely from marking CD45. CD3 and CD68 were used as specific markers for T lymphocytes and macrophages [27], while IL-1β, IL-6 and TNF-α were used as common inflammatory markers [16]. 

For the groups of left knees that received an intra-articular injection, the MP group’s inflammatory reactions (IL-1β, TNF-α, IL-6 and CD68) increased considerably more than those of the other groups. Considering the physical properties of CoCrMo particles, released nanometer-sized particles probably cause physical harm, especially when the knee joint is in motion [21]. This may be one reason why CoCrMo particles had very intensive inflammatory reactions in this in vivo study. Additionally, via various released cellular mediators, damaged cells can lead to the activation and recruitment of immunocompetent cells, especially macrophages. Subsequently, recruited macrophages phagocytize some small CoCrMo particles, while foreign body multinucleated giant cells surround very large particles [44]. Inside the cells, the particles are exposed to oxidative attacks, and consequently, high levels of ions might be released during the chemical corrosion processes [45]. Therefore, CoCrMo particles not only have a destructive effect because of their physical properties—the corrosion process and the presence of metal ions can also cause some biological reactions in the surrounding tissues [46]. Furthermore, because of the presence of phagocytized debris, cellular necrosis can occur and many mediators of inflammation are released by macrophages, which can recruit more macrophages, amplifying the inflammatory cascade [20]. Numerous macrophages (CD68 positive cells) were found in the MP group in this study. Although metal ions can also initiate oxidation-reduction reactions, the metal ions were rapidly quenched, while metal particles offered a reservoir of redox-reactive metals for continuous Reactive oxygen species (ROS) generation, which could further explain why metal particles resulted in the strongest local inflammatory reactions in this study.

However, in contrast with the inflammatory reaction results in our murine model, some in vitro studies have shown different results. Chamaon et al. indicated that the treatment of a human monocytic cell line with ionic cobalt led to a decrease in metabolic activity [Water Soluble Tetrazolium-1(WST-1) assay], while CoCrMo particles had no effect [47]. They explained that the rather large abrasive particles (from 200 nm to several micrometers) used in their study might have resulted in the relatively low impact of CoCrMo particles [47]. Because particle size is considered a critical parameter that influences the biological reactions to wear particles, the majority of MoM-produced particles are usually from 40 to 60 nm [2,48]. One easily neglected factor should also be considered: the in vitro cell cultivation process was static and could not reflect physical injury from metal particles in vivo. Moreover, Caicedo et al. showed that similar increases of IL-1β, TNF-α and IL-6 were evoked by cobalt, molybdenum ions, and Co-Cr-Mo alloy particles in human monocytes/macrophages [49]. Meanwhile, they found that ions and cobalt alloy particles induced inflammasome activation in vitro in a dose-dependent manner [50]. In our murine model, metal ions might be unavoidably systemically disseminated via lymphatics and blood vessels and then circulated throughout the host body, probably leading to a more general distribution than the metal particles. The gradually decreased metal ion level could attenuate local inflammatory stimulation effects, which might be one important reason why the level of inflammatory mediators (IL-1β, IL-6 and TNF-α) and the number of macrophages (CD68+ cells) did not significantly increase in the MI group in our study. 

According to the literature, a cell-mediated (type-IV delayed hypersensitivity) response, which is mainly characterized by the activation of T lymphocytes, can be triggered by metal particles and ions during the process of aseptic loosening [19,51]. At least theoretically, metal ions are able to activate type-IV hypersensitivity by forming haptens with host proteins [16,52]. Compared with toxic reactions, type-IV hypersensitivity reactions do not have a simple dose–response relationship, with higher doses being more potent than lower doses, because even small amounts of antigens can cause strong reactions [17]. In our study, we attempted to count T lymphocytes (CD3-positive cells) to verify the existence of a type-IV hypersensitivity response. However, unexpectedly, regarding T lymphocytes, there were no significant differences among the groups. A delayed hypersensitivity response typically occurred between 24 and 48 h after exposure to an antigen [53]. One possibility was that a delayed-type hypersensitivity response did not play an important role in aseptic implant loosening, and that T lymphocytes were scarce in the primary process. Many researchers support this opinion, because there is a relatively low revision rate related to “allergic” loosening [20]. Approximately 10% of the population is hypersensitive to the materials found in jewelry and joint replacements [54]. If metallic debris and ions can induce a serious hypersensitivity response in the periprosthetic tissues, then more revision surgeries might have been performed as a result of “allergic osteolysis and aseptic loosening”; however, this is not actually the case [20,54]. 

Some studies have indicated that small nanoparticles and high levels of metal ions from implants can be detected in remote organs, including the liver, lung, spleen and kidney [55]. Whether the systemic dissemination of wear particles and metal ions can cause side effects is a matter of debate [20,23]. As such, in our study, the synovial membrane of each right-sided knee joint after the intra-articular particle or ion injection (left knee) was also analyzed for the presence of biological markers (IL-1β, IL-6 TNF-α, CD45, CD68 and CD3). For IL-1β and TNF-α, only the right knees of the MP group had a stronger response than the PBS group, which means that a systemic response caused by metal particles occurred. By contrast, IL-6 was not significantly increased in the right knees of the MP group. One possible explanation for this result is the presence of a temporal factor, because IL-6 secretion in the periprosthetic membrane is preceded by the expression of TNF-α and IL-1β [56]. With regard to CD45, the results of the right knees in the MI group were consistent with those of the left knees, indicating that some immunocompetent cells (CD45-positive cells) were activated by metal ions, and that metal ions also led to systemic reactions. In terms of CD3 and CD68, there were no significant differences in the right knees of all groups, which means that no macrophages or T lymphocytes were recruited in the right knees of all groups. 

There are some limitations to this study. Although the murine model can reflect inflammatory reactions caused by implant materials, it cannot imitate the osteolysis process around implants. Additionally, the single-injection murine model used in this study might not completely reflect the chronic production of wear debris in patients with joint replacements. Some researchers have used osmotic pumps in a murine model to achieve a continuous infusion of degeneration products [57]. This model is more like the clinical scenario, but irritation from osmotic pumps might be an interference factor. In a further study, we will aim to establish one continuous infusion model in combination with existing techniques. To investigate the systemic reactions caused by particles and ions, we used untreated knees. In the future, the impact of metal particles and ions on other organs, such as the liver, spleen, heart and kidney, will also be evaluated, which would further reflect the overall reactions caused by metal debris. Additionally, only one concentration of metal particles and ions was used, referring to the national animal laws. In a future study, different metal particle and ion concentrations, and the impact of different concentrations on aseptic inflammation, will be investigated.

## 5. Conclusions

The results of this study clearly demonstrate that the primary process which occurs after the release of metal particle and ions, especially CoCrMo particles, can lead to an intensive proinflammatory response in vivo. Metal ions can also cause the recruitment of immunocompetent cells but, in view of local inflammatory reactions, macrophages and inflammatory mediators were scarce in vivo. During the process by which metal particles and ions were released in present study, T lymphocytes were not recruited in our murine model. Systemic reactions by metal particles and ions were found according to the observation of untreated right knees.

## Figures and Tables

**Table 1:** Morphological parameters of particles. The CoCr29Mo6 particle shape is predominantly oval and round with a small proportion of acicular particles (shape: round, 44%; oval, 49%; needle, 7%). Equivalent circular diameter (ECD).

Material	ECD	Aspect Ratio	Roundness
CoCr29Mo6 alloy	61.25 ± 18.47nm	1.69 ± 0.66	0.64 ± 0.16

The CoCr29Mo6 particle shape was predominantly oval and round with a small proportion of acicular particles (shape: round, 44%; oval, 49%; needle, 7%).

**Table 2:** Total ion concentrations.

Content in	Co	Cr	Mo	Ni
stock solution	12.0 ± 2.4 mg/L	3.9 ± 0.6 mg/L	0.9 ± 0.1 mg/L	1.3 ± 0.6 mg/L
experimental solution	120 ± 24 µg/L	39 ± 5.7 µg/L	8.8 ± 1.1 µg/L	12.8 ± 6.0 µg/L

Total ion concentrations of the CoCrMo stock solution as well as in the experimental solution (200 µg/L in total) according to Jonitz-Heincke et al. [32].

## References

[1] Eliaz N. (2019). Corrosion of Metallic Biomaterials: A Review. Materials.

[2] Catelas I., Bobyn J.D., Medley J.B., Krygier J.J., Zukor D.J., Huk O.L. (2003). Size, shape, and composition of wear particles from metal-metal hip simulator testing: Effects of alloy and number of loading cycles. J. Biomed. Mater. Res. A.

[3] Merola M., Affatato S. (2019). Materials for Hip Prostheses: A Review of Wear and Loading Considerations. Materials.

[4] Grieco P.W., Pascal S., Newman J.M., Shah N.V., Stroud S.G., Sheth N.P., Maheshwari A.V. (2018). New alternate bearing surfaces in total hip arthroplasty: A review of the current literature. J. Clin. Orthop. Trauma.

[5] Xu J., Yang J., Nyga A., Ehteramyan M., Moraga A., Wu Y., Zeng L., Knight M.M., Shelton J.C. (2018). Cobalt (II) ions and nanoparticles induce macrophage retention by ROS-mediated down-regulation of RhoA expression. Acta Biomater..

[6] Sidaginamale R.P., Joyce T.J., Bowsher J.G., Lord J.K., Avery P.J., Natu S., Nargol A.V., Langton D.J. (2016). The clinical implications of metal debris release from the taper junctions and bearing surfaces of metal-on-metal hip arthroplasty: Joint fluid and blood metal ion concentrations. Bone Jt. J..

[7] Kretzer J.P., Mueller U., Streit M.R., Kiefer H., Sonntag R., Streicher R.M., Reinders J. (2018). Ion release in ceramic bearings for total hip replacement: Results from an in vitro and an in vivo study. Int. Orthop..

[8] Utzschneider S., Paulus A., Datz J.C., Schroeder C., Sievers B., Wegener B., Jansson V. (2009). Influence of design and bearing material on polyethylene wear particle generation in total knee replacement. Acta Biomater..

[9] Pourzal R., Catelas I., Theissmann R., Kaddick C., Fischer A. (2011). Characterization of Wear Particles Generated from CoCrMo Alloy under Sliding Wear Conditions. Wear.

[10] Sansone V., Pagani D., Melato M. (2013). The effects on bone cells of metal ions released from orthopaedic implants. A review. Clin. Cases Miner. Bone Metab..

[11] Yu F.Y., Xie C.Q., Sun J.T., Peng W., Huang X.W. (2018). Overexpressed miR-145 inhibits osteoclastogenesis in RANKL-induced bone marrow-derived macrophages and ovariectomized mice by regulation of Smad3. Life Sci..

[12] Gallo J., Vaculova J., Goodman S.B., Konttinen Y.T., Thyssen J.P. (2014). Contributions of human tissue analysis to understanding the mechanisms of loosening and osteolysis in total hip replacement. Acta Biomater..

[13] Pajarinen J., Jamsen E., Konttinen Y.T., Goodman S.B. (2014). Innate immune reactions in septic and aseptic osteolysis around hip implants. J. Long Term Eff. Med. Implants.

[14] Campbell P., Ebramzadeh E., Nelson S., Takamura K., De Smet K., Amstutz H.C. (2010). Histological features of pseudotumor-like tissues from metal-on-metal hips. Clin. Orthop. Relat. Res..

[15] Mao X., Tay G.H., Godbolt D.B., Crawford R.W. (2012). Pseudotumor in a well-fixed metal-on-polyethylene uncemented hip arthroplasty. J. Arthroplast..

[16] Goodman S.B. (2007). Wear particles, periprosthetic osteolysis and the immune system. Biomaterials.

[17] Granchi D., Savarino L.M., Ciapetti G., Baldini N. (2018). Biological effects of metal degradation in hip arthroplasties. Crit. Rev. Toxicol..

[18] Granchi D., Savarino L., Ciapetti G., Cenni E., Rotini R., Mieti M., Baldini N., Giunti A. (2003). Immunological changes in patients with primary osteoarthritis of the hip after total joint replacement. J. Bone Jt. Surg. Br..

[19] Konttinen Y.T., Pajarinen J. (2013). Adverse reactions to metal-on-metal implants. Nat. Rev. Rheumatol..

[20] Gallo J., Goodman S.B., Konttinen Y.T., Wimmer M.A., Holinka M. (2013). Osteolysis around total knee arthroplasty: A review of pathogenetic mechanisms. Acta Biomater..

[21] Abu-Amer Y., Darwech I., Clohisy J.C. (2007). Aseptic loosening of total joint replacements: Mechanisms underlying osteolysis and potential therapies. Arthritis. Res. Ther..

[22] De Smet K., De Haan R., Calistri A., Campbell P.A., Ebramzadeh E., Pattyn C., Gill H.S. (2008). Metal ion measurement as a diagnostic tool to identify problems with metal-on-metal hip resurfacing. J. Bone Jt. Surg. Am..

[23] Posada O.M., Tate R.J., Grant M.H. (2015). Toxicity of cobalt-chromium nanoparticles released from a resurfacing hip implant and cobalt ions on primary human lymphocytes in vitro. J. Appl. Toxicol..

[24] Lin T.H., Tamaki Y., Pajarinen J., Waters H.A., Woo D.K., Yao Z., Goodman S.B. (2014). Chronic inflammation in biomaterial-induced periprosthetic osteolysis: NF-kappaB as a therapeutic target. Acta Biomater..

[25] Tchilian E.Z., Beverley P.C. (2002). CD45 in memory and disease. Arch. Immunol. Ther. Exp..

[26] Hermiston M.L., Xu Z., Weiss A. (2003). CD45: A critical regulator of signaling thresholds in immune cells. Annu. Rev. Immunol..

[27] Martin-Romero C., Santos-Alvarez J., Goberna R., Sanchez-Margalet V. (2000). Human leptin enhances activation and proliferation of human circulating T lymphocytes. Cell. Immunol..

[28] Xu W., Holzhuter G., Sorg H., Wolter D., Lenz S., Gerber T., Vollmar B. (2009). Early matrix change of a nanostructured bone grafting substitute in the rat. J. Biomed. Mater. Res. B Appl. Biomater..

[29] Ramprasad M.P., Terpstra V., Kondratenko N., Quehenberger O., Steinberg D. (1996). Cell surface expression of mouse macrosialin and human CD68 and their role as macrophage receptors for oxidized low density lipoprotein. Proc. Natl. Acad. Sci. USA.

[30] Reinders J., Sonntag R., Vot L., Gibney C., Nowack M., Kretzer J.P. (2015). Wear testing of moderate activities of daily living using in vivo measured knee joint loading. PLoS ONE.

[31] Schroder C., Reinders J., Zietz C., Utzschneider S., Bader R., Kretzer J.P. (2013). Characterization of polyethylene wear particle: The impact of methodology. Acta Biomater..

[32] Jonitz-Heincke A., Tillmann J., Klinder A., Krueger S., Kretzer J.P., Hol P.J., Paulus A.C., Bader R. (2017). The Impact of Metal Ion Exposure on the Cellular Behavior of Human Osteoblasts and PBMCs: In Vitro Analyses of Osteolytic Processes. Materials.

[33] Holt G., Murnaghan C., Reilly J., Meek R.M. (2007). The biology of aseptic osteolysis. Clin. Orthop. Relat. Res..

[34] Al Saffar N., Revell P.A. (1994). Interleukin-1 production by activated macrophages surrounding loosened orthopaedic implants: A potential role in osteolysis. Br. J. Rheumatol..

[35] Utzschneider S., Becker F., Grupp T.M., Sievers B., Paulus A., Gottschalk O., Jansson V. (2010). Inflammatory response against different carbon fiber-reinforced PEEK wear particles compared with UHMWPE in vivo. Acta Biomater..

[36] Smith A.J., Dieppe P., Vernon K., Porter M., Blom A.W. (2012). Failure rates of stemmed metal-on-metal hip replacements: Analysis of data from the National Joint Registry of England and Wales. Lancet.

[37] Lorber V., Paulus A.C., Buschmann A., Schmitt B., Grupp T.M., Jansson V., Utzschneider S. (2014). Elevated cytokine expression of different PEEK wear particles compared to UHMWPE in vivo. J. Mater. Sci. Mater. Med..

[38] Paulus A.C., Frenzel J., Ficklscherer A., Rossbach B.P., Melcher C., Jansson V., Utzschneider S. (2014). Polyethylene wear particles induce TLR 2 upregulation in the synovial layer of mice. J. Mater. Sci. Mater. Med..

[39] Utzschneider S., Lorber V., Dedic M., Paulus A.C., Schroder C., Gottschalk O., Schmitt-Sody M., Jansson V. (2014). Biological activity and migration of wear particles in the knee joint: An in vivo comparison of six different polyethylene materials. J. Mater. Sci. Mater. Med..

[40] Klinder A., Seyfarth A., Hansmann D., Bader R., Jonitz-Heincke A. (2018). Inflammatory Response of Human Peripheral Blood Mononuclear Cells and Osteoblasts Incubated With Metallic and Ceramic Submicron Particles. Front. Immunol..

[41] Kraft C.N., Diedrich O., Burian B., Schmitt O., Wimmer M.A. (2003). Microvascular response of striated muscle to metal debris. A comparative in vivo study with titanium and stainless steel. J. Bone Jt. Surg. Br..

[42] Anabtawi M., Beck P., Lemons J. (2008). Biocompatibility testing of simulated total joint arthoplasty articulation debris. J. Biomed. Mater. Res. B Appl. Biomater..

[43] Tchilian E.Z., Beverley P.C. (2006). Altered CD45 expression and disease. Trends Immunol..

[44] Bijukumar D.R., Segu A., Souza J.C., Li X., Barba M., Mercuri L.G., Jacobs J.J., Mathew M.T. (2018). Systemic and local toxicity of metal debris released from hip prostheses: A review of experimental approaches. Nanomedicine.

[45] Delgado-Ruiz R., Romanos G. (2018). Potential Causes of Titanium Particle and Ion Release in Implant Dentistry: A Systematic Review. Int. J. Mol. Sci..

[46] Haeri M., Wllert T., Langford G.M., Gilbert J.L. (2012). Electrochemical control of cell death by reduction-induced intrinsic apoptosis and oxidation-induced necrosis on CoCrMo alloy in vitro. Biomaterials.

[47] Chamaon K., Schonfeld P., Awiszus F., Bertrand J., Lohmann C.H. (2019). Ionic cobalt but not metal particles induces ROS generation in immune cells in vitro. J. Biomed. Mater. Res. B Appl. Biomater..

[48] Hallab N.J., Jacobs J.J. (2009). Biologic effects of implant debris. Bull. NYU Hosp. Jt. Dis..

[49] Caicedo M.S., Pennekamp P.H., McAllister K., Jacobs J.J., Hallab N.J. (2010). Soluble ions more than particulate cobalt-alloy implant debris induce monocyte costimulatory molecule expression and release of proinflammatory cytokines critical to metal-induced lymphocyte reactivity. J. Biomed. Mater. Res. A.

[50] Caicedo M.S., Desai R., McAllister K., Reddy A., Jacobs J.J., Hallab N.J. (2009). Soluble and particulate Co-Cr-Mo alloy implant metals activate the inflammasome danger signaling pathway in human macrophages: A novel mechanism for implant debris reactivity. J. Orthop. Res..

[51] Pandit H., Vlychou M., Whitwell D., Crook D., Luqmani R., Ostlere S., Murray D.W., Athanasou N.A. (2008). Necrotic granulomatous pseudotumours in bilateral resurfacing hip arthoplasties: Evidence for a type IV immune response. Virchows Arch. Int. J. Pathol..

[52] Dapunt U., Giese T., Prior B., Gaida M.M., Hansch G.M. (2014). Infectious versus non-infectious loosening of implants: Activation of T lymphocytes differentiates between the two entities. Int. Orthop..

[53] Warrington R., Watson W., Kim H.L., Antonetti F.R. (2011). An introduction to immunology and immunopathology. Allergy Asthma Clin. Immunol..

[54] Burkandt A., Katzer A., Thaler K., Von Baehr V., Friedrich R.E., Ruther W., Amling M., Zustin J. (2011). Proliferation of the synovial lining cell layer in suggested metal hypersensitivity. In Vivo.

[55] Urban R.M., Tomlinson M.J., Hall D.J., Jacobs J.J. (2004). Accumulation in liver and spleen of metal particles generated at nonbearing surfaces in hip arthroplasty. J. Arthroplast..

[56] Dinarello C.A. (2005). Blocking IL-1 in systemic inflammation. J. Exp. Med..

[57] Ma T., Huang Z., Ren P.G., McCally R., Lindsey D., Smith R.L., Goodman S.B. (2008). An in vivo murine model of continuous intramedullary infusion of polyethylene particles. Biomaterials.

